# Long-term deep brain stimulation of the ventral anterior limb of the internal capsule for treatment-resistant depression

**DOI:** 10.1136/jnnp-2019-321758

**Published:** 2019-12-04

**Authors:** Junus M. van der Wal, Isidoor O. Bergfeld, Anja Lok, Mariska Mantione, Martijn Figee, Peter Notten, Guus Beute, Ferdinand Horst, Pepijn van den Munckhof, P. Rick Schuurman, Damiaan Denys

**Affiliations:** 1 Psychiatry, Amsterdam UMC - Locatie AMC, Amsterdam, Noord-Holland, The Netherlands; 2 Amsterdam Brain and Cognition, University of Amsterdam, Amsterdam, Noord-Holland, The Netherlands; 3 Neurology, University Medical Center Utrecht, Utrecht, The Netherlands; 4 Psychiatry, Icahn School of Medicine at Mount Sinai, New York, New York, USA; 5 Psychiatry, Elisabeth-TweeSteden Ziekenhuis, Tilburg, Noord-Brabant, The Netherlands; 6 Neurosurgery, Elisabeth-TweeSteden Ziekenhuis, Tilburg, Noord-Brabant, The Netherlands; 7 Neurosurgery, Amsterdam UMC - Locatie AMC, Amsterdam, Noord-Holland, The Netherlands; 8 Netherlands Institute for Neuroscience, Amsterdam, Noord-Holland, The Netherlands

## Abstract

**Objective:**

Deep brain stimulation (DBS) reduces depressive symptoms in approximately 40%–60% of patients with treatment-resistant depression (TRD), but data on long-term efficacy and safety are scarce. Our objective was to assess the efficacy and safety of DBS targeted at the ventral anterior limb of the internal capsule (vALIC) in 25 patients with TRD during a 1-year, open-label, maintenance period, which followed a 1-year optimisation period.

**Methods:**

Depression severity was measured using the 17-item Hamilton Depression Rating Scale (HAM-D-17), Montgomery-Asberg Depression Rating Scale (MADRS) and self-reported Inventory of Depressive Symptomatology (IDS-SR). Primary outcomes were response rate (≥50% HAM-D-17 score reduction) after the maintenance phase, approximately 2 years after DBS surgery, and changes in depression scores and occurrence of adverse events during the maintenance phase.

**Results:**

Of 25 operated patients, 21 entered and 18 completed the maintenance phase. After the maintenance phase, eight patients were classified as responder (observed response rate: 44.4%; intention-to-treat: 32.0%). During the maintenance phase, HAM-D-17 and MADRS scores did not change, but the mean IDS-SR score decreased from 38.8 (95% CI 31.2 to 46.5) to 35.0 (95% CI 26.1 to 43.8) (p=0.008). Non-responders after optimisation did not improve during the maintenance phase. Four non-DBS-related serious adverse events occurred, including one suicide attempt.

**Conclusions:**

vALIC DBS for TRD showed continued efficacy 2 years after surgery, with symptoms remaining stable after optimisation as rated by clinicians and with patient ratings improving. This supports DBS as a viable treatment option for patients with TRD.

**Trial registration number:**

NTR2118.

## Introduction

Depression is a highly prevalent psychiatric disorder affecting over 300 million people worldwide in 2015.^1^ Globally, it is the leading cause of disability and a major risk factor for suicide, resulting in approximately 800 000 deaths annually.^1^ Despite decades of research aiming to optimise treatment, up to 30% of patients fail to respond to multiple treatment steps consisting of pharmacological and/or psychotherapeutic interventions.^2^ In addition, medication failure is associated with poorer response to subsequent electroconvulsive therapy (ECT), with non-response estimated to be as high as 52% in this group.^3 4^ Typically, patients who do not respond to two or more treatment steps are considered to suffer from treatment-resistant depression (TRD).^5^ Patients with TRD show higher rates of psychosocial stress, hospitalisation and suicide than non-resistant patients, resulting in high disease and societal burden.^6 7^

For patients with TRD, deep brain stimulation (DBS) is currently an experimental treatment, with which pathological neuronal activity in specific brain targets is modulated by electrical stimulation through implanted electrodes.^8^ DBS targeted to the subcallosal cingulate region, the medial forebrain bundle (MFB) or striatal/capsular areas has been shown to be an effective and safe treatment option for patients with TRD.^9^ Our research group previously studied DBS of the ventral anterior limb of the internal capsule (vALIC), which resulted in a response (ie, ≥50% symptom reduction) in 10 of 25 patients with TRD after an optimisation period with a maximum of 1 year. Moreover, active DBS was significantly more effective than sham DBS.^10^

However, further studies are needed to add to a limited body of data on long-term efficacy and safety of DBS, particularly since relapse rates of conventional treatments for TRD are notoriously high. Indeed, patients receiving treatment as usual (TAU), including ECT, show a disappointing response rate of around 20% after 2 years.^11^ After 2 years or more of DBS, intention-to-treat response rates ranged from 23.3% to 71.4% in 48 patients with DBS targeted at the striatal/capsular area,^12–14^ and from 25.0% to 64.7% in 105 patients with DBS targeted at the subcallosal cingulate region.^15–18^

Here, we report on the follow-up of patients of our study group of vALIC DBS during the 1-year maintenance phase, which followed optimisation.^10^ Our aims were to assess the response rate to DBS after the maintenance phase, approximately 2 years after DBS implantation, and analyse changes in depression severity and record occurrence of adverse events (AEs) during this period. In line with previous studies, we expected to find a fairly stable clinical response.^12–18^

## Patients and methods

### Patients

This study reports on the clinical status during a 1-year maintenance period of 25 patients with TRD recruited at two hospitals in the Netherlands who received vALIC DBS, of which data on the optimisation period and of a double-blind, sham-controlled, cross-over period have been published earlier.^10^

Detailed information on the inclusion and exclusion criteria and the surgical procedure has been described previously.^10^ In short, patients were eligible if they met the criteria for major depressive disorder (MDD) in accordance with the fourth edition of the *Diagnostic and Statistical Manual of Mental Disorders*, with a 17-item Hamilton Depression Rating Scale (HAM-D-17) score of 18 or higher.^19 20^ Additionally, patients were eligible if they failed to respond to at least two classes of second-generation antidepressants in adequate dosage, one trial of tricyclic antidepressant with subsequent lithium augmentation, one trial of monoamine oxidase inhibitors, and six or more sessions of bilateral ECT or relapsed after discontinuation of maintenance ECT. Exclusion criteria were an organic cause of depression, bipolar disorder, schizophrenia or a history of psychosis unrelated to MDD, substance abuse during the past 6 months, antisocial personality disorder, dementia, tic disorder, Parkinson’s disease, epilepsy, unstable physical condition, pregnancy, or general contraindications for surgery.

### DBS treatment and design of the study

**Figure 1:**
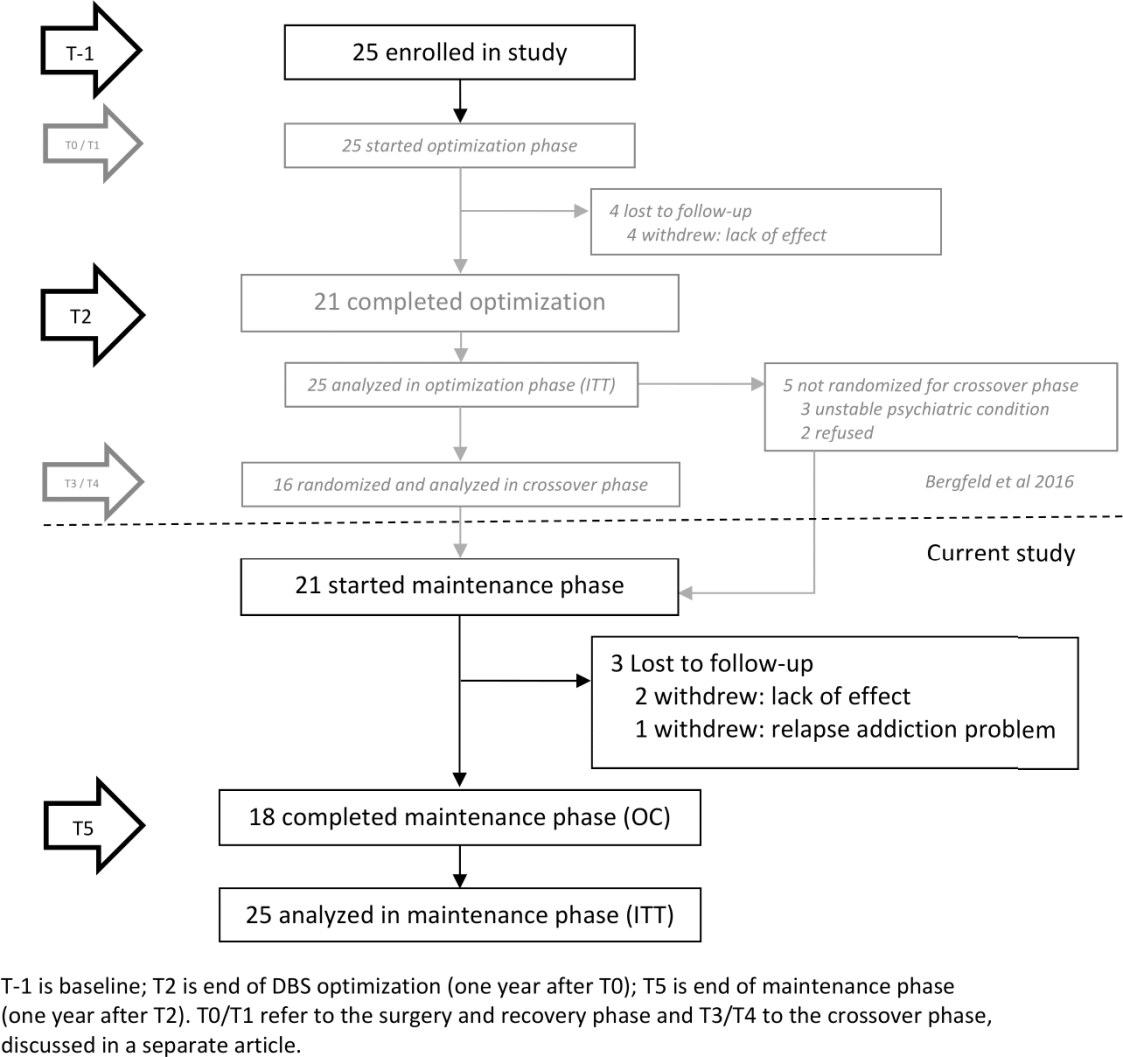
Flow chart of study timeline and patient follow-up throughout the study period. DBS, deep brain stimulation; ITT, intention-to-treat; OC, observed-case.

All patients were implanted bilaterally with four-contact electrodes (lead model 3389; Medtronic), with the lowest contact point in the nucleus accumbens and the three upper contact points in the vALIC. Subcutaneous extensions connected the electrodes to a neurostimulator (Activa PC/RC; Medtronic) placed in the infraclavicular region. After surgery, patients entered an open-label optimisation phase which lasted a maximum of 1 year, followed by a double-blind, sham-controlled, cross-over phase.^10^ Subsequently, patients entered an open-label maintenance phase during which a psychologist or psychiatrist assessed the clinical status of the patients and the occurrence of AEs at least once every 6 months during visits at the outpatient clinic or more frequently depending on clinical status. During this phase, DBS settings were evaluated and further adjusted if necessary, and medication changes or psychotherapy was initiated on indication. The end of the maintenance phase (T5) was 1 year after the end of the optimisation phase (T2) and approximately 2 years after DBS surgery (T0). An overview of the design of the entire study is presented in figure 1.

### Outcome variables

Clinical outcome was measured by HAM-D-17 (range 0–52),^20^ Montgomery-Asberg Depression Rating Scale (MADRS, range 0–60)^21^ and the self-reported Inventory of Depressive Symptomatology (IDS-SR, range 0–84).^22^ A higher score indicates more severe symptoms on all rating scales.

The primary outcomes of this study were response rate at T5 and change in depression scores and occurrence of AEs during the maintenance phase. At T5, response was defined as ≥50% decrease in HAM-D-17 score compared with presurgical baseline score, and remission as HAM-D-17 score ≤7.^23^ Furthermore, to provide a more detailed description of the clinical course of the patients during the maintenance phase, patients were subcategorised at T2 and T5 based on HAM-D-17 reduction as strong (≥75% HAM-D-17 reduction), clear (≥50%, but <75%), partial (≥25%, but <50%) or minimal-improvers/responders (<25%). Severity of AEs and their relation to either surgery, device or stimulation were assessed by a psychologist. AEs were considered serious if they resulted in death, life-threatening situations, (prolonged) hospitalisation or chronic disability. We only report on AEs that occurred during the maintenance phase, as AEs during optimisation have been described previously.^10^ The secondary outcome was change in depression scores between baseline and end of maintenance.

### Statistical analysis

The percentage of responders and non-responders is presented descriptively, both on an observed-case (OC) and intention-to-treat (ITT) level, based on the percentage of reduction on the HAM-D-17 score, relative to baseline. In case of premature dropout, last HAM-D-17 observation was carried forward to define the response status. We used three restricted, maximum-likelihood, linear mixed models to test for change in depression scores during the maintenance phase. The dependent variable was depression score (HAM-D-17, MADRS or IDS-SR), and the independent random variable was days since start of the maintenance phase with individual patients as grouping variable. To test for change from baseline, we executed the same models, except for log-transformed days from baseline which were used as an independent variable. Days from baseline were log-transformed to meet assumptions of linearity and normality of residuals.

In a post-hoc analysis, response status at T2 in interaction with time was added as fixed independent variable to the mixed models, to test for differences between initial responders and non-responders. We set α to 0.0167 (Bonferroni-corrected for the number of scales: 0.05/3). We report p values between 0.0167 and 0.05 as trends. All analyses were performed using IBM SPSS Statistics V.24.^24^

## Results

Of the 25 patients treated with DBS, 21 entered and 18 completed the maintenance phase (see figure 1 for a flow chart outlining the follow-up and reasons for dropout). Demographic and clinical characteristics and follow-up information of both the initially enrolled patients as well as the patients who entered the maintenance phase are described in table 1. The mean follow-up time of all patients after surgery was 99.4 weeks (SD: 39.2 weeks). On average, the maintenance phase lasted 62.4 weeks (SD: 15.6 weeks), with a median number of patient visits of 7 (range: 2–49).

**Table 1:** Descriptive characteristics of study participants

	Enrolled in study and analysed in ITT (n=25)	Started maintenance phase (n=21)
Sex		
Male, n (%)	8 (32)	6 (29)
Female, n (%)	17 (68)	15 (71)
Age (years) at inclusion, mean (SD)	53.2 (8.4)	52.5 (8.6)
Age (years) at TRD onset, mean (SD)		
Self-report	28.5 (15.2)	28.0 (15.0)
Diagnosis	37.8 (9.8)	37.3 (9.9)
Years since TRD onset (diagnosis), mean (SD)	15.3 (9.1)	15.1 (8.5)
Estimated IQ, mean (SD)	95.3 (15.0)	95.6 (14.9)
Number of past medications, mean (SD)	10.8 (3.3)	10.9 (3.2)
Number of past ECT series, mean (SD)	2.3 (1.7)	2.2 (1.4)
Number of past ECT sessions, mean (SD)	68.9 (103.6)	70.7 (112.1)
Patients with past suicide attempts, n (%)	7 (28)	6 (29)
TRD episodes, n (%)		
1	10 (40)	10 (48)
2	3 (12)	3 (14)
>2	12 (48)	8 (38)
Duration of current episode, months (SD)	83.8 (76.2)	89.0 (81.1)
Hospital		
Academic Medical Center	12	11
St Elisabeth Hospital	13	10
Duration of follow-up (weeks), range (mean, SD)	20.9–154.0 (99.4, 39.2)	56∙6–154.0 (112.0, 28.1)
Duration of maintenance phase (weeks), range (mean, SD)	–	14.0–91.4 (62.4, 15.6)*
Number of outpatient clinic visits during maintenance phase, range (median)	–	2–49 (7)*

*In these descriptive analyses, two patients were excluded with measurements at T2 but no further follow-up during the maintenance phase due to withdrawal from study (lack of effect).

ECT, electroconvulsive therapy; ITT, intention-to-treat; T2, start of the maintenance phase; TRD, treatment-resistant depression.

**Figure 2:**
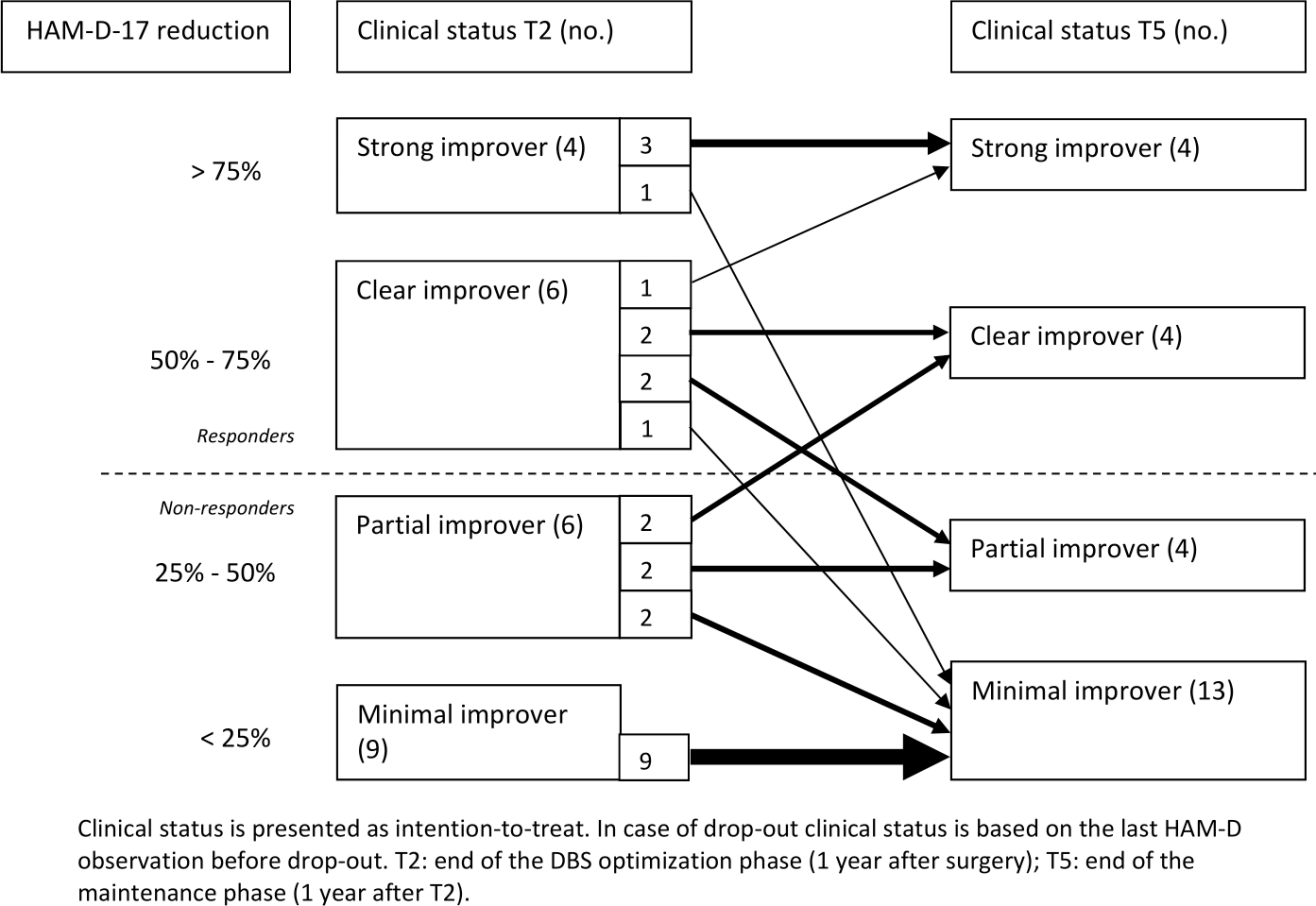
Clinical status of patients before and after the maintenance phase. DBS, deep brain stimulation; HAM-D-17, 17-item Hamilton Depression Rating Scale.

An overview of patients’ response and improvement status at T2 and T5 is shown in figure 2 (all ITT). Of the 18 patients who completed the maintenance phase, 8 (44.4% OC or 32.0% ITT) were classified as a responder at the end of the study, 5 of whom remitted (OC: 27.8%; ITT: 20.0%). Of the 10 initial responders at T2, 6 were still a responder at T5. One of these patients, a strong improver at both T2 and T5, experienced a 27-week relapse of symptoms during the maintenance phase due to battery depletion. The other five patients showed a stable response throughout the maintenance phase. Two initial partial improvers at T2 reached the response threshold during the maintenance phase and were classified as responder at T5. For one of these patients, T5 was the first time during the study the response threshold was reached; the other patient reached the response threshold only once before, during the optimisation phase. In the ITT analysis, all minimal improvers at T2 remained minimal improver at T5.

The mean, SD and 95% CI of all depression scores at baseline, T2 and T5 are displayed in table 2. The mean HAM-D-17 score changed from 15.0 (95% CI 11.4 to 18.6) at T2 to 16.6 (12.2 to 20.9) at T5, MADRS from 22.9 (17.7 to 28.1) at T2 to 24.3 (18.1 to 30.5) at T5, and IDS-SR from 38.8 (31.2 to 46.5) at T2 to 35.0 (26.1 to 43.8) at T5.

**Table 2:** Depression scores at baseline and before and after the maintenance phase

	**Baseline**			**T2**			**T5**		
	**N**	**Mean, SD**	**95%** CI	**N**	**Mean, SD**	**95%** CI	**N**	**Mean, SD**	**95%** CI
All patients									
HAM-D-17 (ITT)	25	22.2, 4.9	20.2–24.2	25	15.0, 8.7	11.4–18.6	25	16.6, 10.5	12.2–20.9
HAM-D-17 (OC)				21	14.5, 8.6	10.6–18.4	18	13.6, 9.2	9.0–18.2
MADRS (ITT)	25	34.0, 5.8	31.6–36.5	25	22.9, 12.7	17.7–28.1	25	24.3, 15.1	18.1–30.5
MADRS (OC)				21	21.8, 12.9	15.9–27.6	18	19.8, 14.4	12.7–27.0
IDS-SR (ITT)	25	49.4, 9.8	45.3–53.4	25	38.8, 18.4	31.2–46.5*	25	35.0, 21.4	26.1–43.8*
IDS-SR (OC)				21	38.0, 19.0	29.3–46.6	18	29.9, 20.3	19.8–40.0
Responders†									
HAM-D-17	10	21.5, 6.1	17.1–25.9	10	8.0, 4.7	4.7–11.3	8	5.5, 5.0	1.3–9.8
MADRS	10	31.2, 6.5	26.6–35.9	10	11.8, 7.4	6.5–17.11	8	8.3, 8.5	1.2–15.3
IDS-SR	10	42.0, 9.4	35.3–48.7	10	23.2, 12.0	14.6–31.8	8	14.0, 9.7	5.9–22.1
Non-responders†									
HAM-D-17 (ITT)	15	22.6, 4.0	20.4–24.8	15	19.7, 7.6	15.5–23.9	17	21.8, 7.9	17.7–25.8
HAM-D-17 (OC)				11	20.5, 6.8	15.9–25.0	10	20.1, 6.0	15.8–24.4
MADRS (ITT)	15	35.9, 4.7	33.4–38.5	15	31.3, 9.1	26.0–36.5	17	31.8, 10.9	26.2–37.4
MADRS (OC)				11	30.8, 9.6	24.4–37.3	10	29.1, 11.1	21.2–37.1
IDS-SR (ITT)	15	54.2, 6.7	50.6–58.0	15	49.3, 14.1	41.5–57.1	17	44.8, 18.0	35.6–54.1
IDS-SR (OC)				11	51.4, 13.4	42.4–60.4	10	42.6, 17.5	30.1–55.1

*Difference from T2 to T5: p=0.008

†Response status based on HAM-D-17 score at T2 (baseline and T2) or T5 (T5), NB: OC and ITT values for responders are similar.

HAM-D-17, 17-item Hamilton Depression Rating Scale; IDS-SR, self-reported Inventory of Depressive Symptomatology;ITT, intention-to-treat; MADRS, Montgomery-Asberg Depression Rating Scale; OC, observed-case; T2, start of the maintenance phase; T5, end of the maintenance phase.

The mixed model analysis showed that change over time during the maintenance phase for the entire group was not significant for the HAM-D-17 (*F*(1, 17.3)=0.9, p=0.35) or MADRS (*F*(1, 18.5)=0.7, p=0.41) scores. However, the IDS-SR score in the entire group showed a significant decrease during this period (*F*(1, 17.4)=9.1, p=0.008). Online supplementary figure S1 graphically illustrates this decrease by displaying the course of HAM-D-17 and IDS-SR score during the maintenance phase, for responders and non-responders separately.

10.1136/jnnp-2019-321758.supp1 Supplementary data

In addition, for the entire group, HAM-D-17 (*F*(1, 20.7)=13.7, p=0.001) and MADRS (*F*(1, 20.7)=13.7, p=0.001) scores had significantly decreased over time from baseline until the end of the maintenance phase, whereas IDS-SR scores (*F*(1, 20.7)=6.22, p=0.02) showed a trend towards decrease when measured throughout this period. Post-hoc analysis showed there was no significant difference in the course of depression scores during the maintenance phase between initial responders and non-responders (HAM-D-17: *F*(1, 15.7)=0.20, p=0.66; MADRS: *F*(1, 17.1)=2.15, p=0.16; IDS-SR: *F*(1, 14.5)=1.55, p=0.23).

Table 3 lists all AEs that occurred during the maintenance phase and their relation to DBS. There were four serious AEs in three patients during the maintenance phase, none of which could be reliably linked to DBS. One non-responder attempted suicide using medication during the maintenance phase, in addition to the four patients who attempted suicide during the optimisation period. One patient, a responder who experienced relapse of depressive symptoms during the maintenance phase due to battery depletion, developed increase in depressive symptoms and suicidal ideations and took several months after battery replacement to regain full response. Furthermore, one non-responder committed an autointoxication using medication without intent to commit suicide.

**Table 3:** Adverse events during maintenance phase

	Duration of effect	Patients/reports (n)
Device-related		
Pain around extensions	Transient	1/1
Pain around burr holes	Transient	1/1
Palpitations around neurostimulator	Transient	1/1
Stimulation-related		
Nausea	Transient	2/4
Anxiety	Transient	1/3
Flight of ideas	Transient	1/1
Agitation	Transient	3/5
Muscle cramp	Transient	1/1
Headache	Transient	1/1
Blurred vision	Transient	1/2
Dizziness	Transient	1/3
Unknown		
Change in taste	Permanent	1/1
Change in taste	Transient	1/2
Agitation	Transient	4/4
Memory disturbance	Transient	2/2
Suicidal ideation	Transient	6/7
Paraesthesia	Transient	2/2
Palpitations	Transient	2/5
Complaints of limbs	Transient	4/15
Blurred vision	Transient	3/7
Headache	Transient	4/14
Chest pain	Transient	1/2
Neck pain	Transient	1/3
Increased depressive thoughts	Transient	1/2 (serious adverse event)
Excessive sweating	Transient	3/6
Diarrhoea	Transient	1/1
Stomach aches	Transient	2/5
Abnormal body temperature	Transient	3/10
Panic	Transient	1/1
Dyspnoea	Transient	1/2
Chest tightness	Transient	1/2
Fatigue	Transient	4/8
Restlessness	Transient	1/3
Joint pain	Transient	1/2
Nausea	Transient	3/4
Emotionally unstable	Transient	1/2
Sleep disturbances	Transient	2/3
Muscle ache	Transient	2/3
Concentration disturbance	Transient	1/1
Dry mouth	Transient	2/2
Salivation	Transient	1/1
Pain in mouth	Transient	1/1
Constipation	Transient	1/2
Hopelessness	Transient	1/1
Intestinal aches	Transient	1/1
Suicide attempt	Transient	1/1 (serious adverse event)
Pain in eyes	Transient	1/1
Dizziness	Transient	1/1
Increased sensory sensitivity	Transient	1/1
Confusion	Transient	1/1
Autointoxication	Transient	1/1 (serious adverse event)
Hypertension	Transient	1/1
Automutilation	Transient	1/1
Recurrence in depression	Transient	1/1

DBS parameters at T2 and T5 are shown in online supplementary table 1. From T2 to T5, we tested a minimal number of DBS settings in strong and clear responders (median: 2, mean: 4.9, SD: 5.0) and partial responders (median: 2, mean: 5.6, SD: 5.7). In non-responders, the number of tested settings was considerably higher (median: 16, mean: 20.0, SD: 17.5). In general, we tried minor increases in amplitude and pulse width to see whether additional response could be achieved. In addition, lowering voltage or pulse width in some patients yielded the same effect, but resulted in a longer battery life. An overview of medication use among patients is presented in online supplementary table 2. Change in medication during the maintenance phase took place in eight patients. Changes in responders were an increase in benzodiazepines (n=1), decrease in benzodiazepines alone (n=1), or together with antipsychotics (n=1). Changes in non-responders (n=5) reflected mostly a change in pharmacological treatment strategy.

## Discussion

We found vALIC DBS to be effective in 32% of patients with TRD (ITT) after approximately 2 years of follow-up compared with 40% after optimisation, with no significant changes in average depression severity during a 1-year maintenance phase. However, subjective symptoms (IDS-SR) significantly improved between 1 and 2 years. Most patients showed a stable clinical response to DBS during long-term follow-up and tolerated the treatment well. Notably, all patients who did not or only minimally improved in the first year of treatment also did not improve during the second year.

Our response rate is in line with results of three other studies on striatal/capsular DBS with a total of 48 patients (range 7–30) showing an average response rate of 35.3% (range 23.3%–71.4%) after a follow-up period of 2 or 3 years after surgery.^12–14^ Our results are also comparable with response rates found following DBS of the subcallosal cingulate region, in which four studies with a total of 105 patients (range 8–60) found an average response rate of 39.3% (range 25.0%–64.7%) 2 years after surgery.^15–18^ Response rates in the first patients treated with MFB DBS are higher: 5 years after surgery, 8 of 11 patients showed a full response, although an additional 5 patients had dropped out due to non-response at that point.^25^ Irrespective of target, these response rates are all higher than those of patients with TRD receiving TAU, which are around 20%.^11^ This supports DBS as a possible valid treatment step in patients with TRD. Of additional interest is whether DBS could be an alternative for patients currently treated with maintenance ECT, as this treatment poses a large physical (and practical) burden on patients.

Besides the continued effectiveness, this study shows the response is fairly stable over time, given the unchanged HAM-D-17 and MADRS scores during the maintenance phase. However, this was not necessarily reflected by the response status of the patients, as six patients crossed the response threshold during the maintenance phase, either in positive or in negative direction. In some cases, change in response status was evident, such as in the case of an initial strong improver who became minimal improver due to relapse in substance abuse and subsequent increase of depressive symptoms. However, more often than not, depression scores fluctuated around a predefined response threshold (eg, between 45% and 55%), causing considerable changes in categorically defined study endpoints (eg, from non-responder to responder). Indeed, of the four initial responders who were deemed non-responder after the maintenance phase, all but the patient with relapse in substance abuse remained stable on the IDS-SR scale during this period. This shows that a 50% symptom reduction at a fixed time point might not be an optimal definition of response in TRD studies. A possible alternative definition of response should reflect the clinical course and symptomatology of a patient throughout a study period, as opposed to mark symptom reduction at a fixed point in time, such as recently done by Bewernick *et al*.^26^ Furthermore, such an approach could be more in line with recent insights into the dynamics of symptoms in mental disorders.^27^

Interestingly, the patient-rated IDS-SR score decreased further during the maintenance phase, while the clinician-rated HAM-D-17 and MADRS scores did not. Possibly, the IDS-SR is more sensitive to symptom change due to the wider range of questions, parallel to the clinician-rated IDS-C rating scale.^28^ Alternatively, some authors have suggested that self-reported rating scales can ‘lag behind’ on clinician-rated scales during initial improvement, due to a negative cognitive bias which causes patients to over-rate their symptoms.^29^

Importantly, none of the patients who had no or only minimal improvement after 1 year improved during the second year, despite ongoing efforts to optimise DBS parameters. This raises the question to what extent it is opportune to continue DBS treatment in this group. Discontinuing DBS should, however, be weighed with the despair this might cause in patients, as DBS is usually a last resort treatment. Manifold factors could underlie non-response in this group, ranging from patient factors such as personality traits and psychiatric comorbidity, to clinical factors such as inaccurate diagnosis or suboptimal DBS targeting. Compiling data specifically on the course and characteristics of patients with TRD who do not respond and/or choose to discontinue DBS could add to the limited knowledge on factors associated with non-response and clinical course after removal. These data could serve clinicians and patients faced with possible discontinuation of DBS treatment to make more informed decisions.

In most patients, vALIC DBS was well tolerated. Most AEs could not be reliably attributed to effects of stimulation, the in situ device or the operation. This was also the case for all serious AEs that took place during the maintenance phase. One persistent non-responder attempted suicide during the maintenance phase. DBS was activated at the time of the attempt, but stimulation parameters had been stable for months and no recent changes in medication or psychotherapy were made. However, suicidality should be recorded carefully, especially since suicide rates are known to be higher among patients with TRD than patients with a non-resistant depression. Furthermore, non-response to a last resort treatment such as DBS might increase hopelessness and suicidality, although data on the impact of DBS non-response on suicide risk are currently limited.^30^ Furthermore, in one of the responders, battery depletion led to an abrupt increase in depressive symptoms and suicidal ideation requiring hospitalisation. After battery replacement a few weeks later, it took several months to regain full response. The abrupt symptom increase was similar as seen after turning DBS off in the sham-controlled, cross-over phase earlier in the study, although the response was quickly regained after reactivating the DBS in this phase in all patients. The fact that battery replacement took place several weeks after depletion might have contributed to the longer time it took to regain response. This event not only shows discontinuation of stimulation can result in relapse of depressive symptoms even after prolonged period of response, but also stresses the need for timely battery replacement.

This study has limitations that require consideration. First of all, since there was no control group, it could not be determined whether vALIC DBS was superior to TAU in this group. However, as noted earlier, our response rate is higher than the 20% response in a large prospective study in patients with TRD receiving TAU.^29^ Second, change in medication took place in eight patients during the maintenance phase. Nevertheless, response and improvement status were similar for these patients at T2 and T5, so these changes are not expected to have played a major role in the results of this study.

In conclusion, this study shows the effectiveness and safety of vALIC DBS for TRD in the long term and a fairly stable response over time. These results provide further support for vALIC DBS as a treatment option for patients with TRD. Future research should establish the role of DBS in relation to current conventional treatments, especially maintenance ECT.
