# Supplementary material for: Long-term deep brain stimulation of the ventral anterior limb of the internal capsule for treatment-resistant depression
Source: J Neurol Neurosurg Psychiatry. 2019 Dec 4;91(2):189–95. doi: 10.1136/jnnp-2019-321758 (PMC6996094; doi:10.1136/jnnp-2019-321758)
Supplement: Supplementary data [file jnnp-2019-321758supp001.pdf]

Supplementary information

Long-term deep brain stimulation of the ventral anterior limb of the internal capsule for treatment-resistant depression

Figure S1: graphical display of the course of HAM-D-17 and IDS-SR score during the maintenance phase

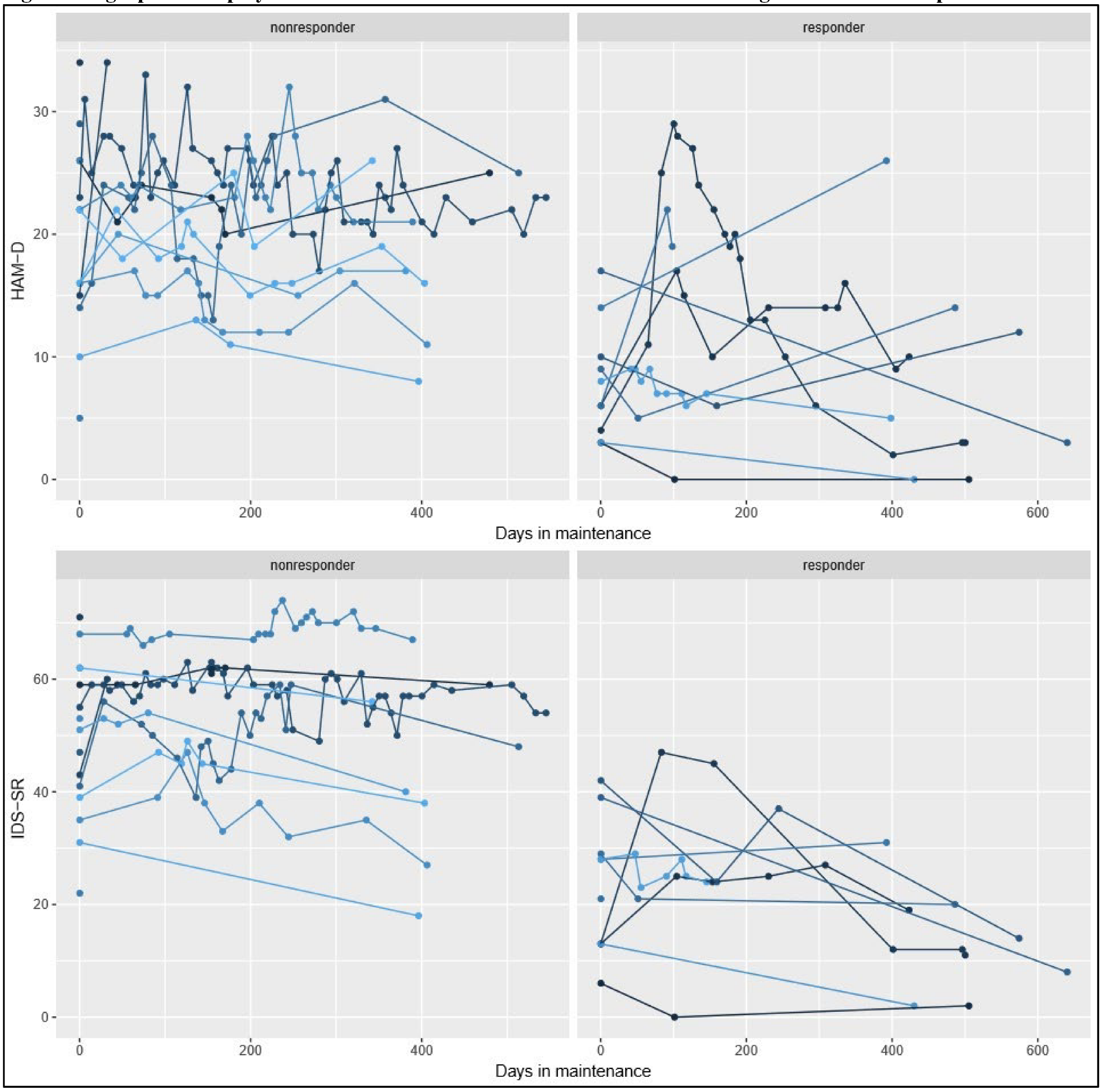

Abbreviations: HAM-D=Hamilton Depression Rating Scale (17 items); IDS-SR-Inventory of Depressive Symptoms, Self-Report.

**Table S1: DBS parameters at T2 and T5**				
**Responders T2 (n = 10)**				
	**T2**		**T5**	
	L	*R*	*L*	*R*
**Voltage (mean, SD)**	5.2, 1.3	5.3, 1.3	5.7, 1.5	5.6, 1.6
**Pulse width (count)**				
90	8	8	6	6
120	2	2	4	4
**Frequency (count)**				
130	1	1	3	3
180	9	9	7	7
**Non-responders T2 (n = 15)**				
	**T2**		**T5**	
	L	*R*	*L*	*R*
**Voltage (mean, SD)**	4.3, 1.1	4.2, 1.1	4.5, 2.0	4.5, 2.0
**Pulse width (count)**				
60	4	4	6	6
90	9	9	7	7
120	1	1	1	1
150	1	1	1	1
**Frequency (count)**				
30	1	1		
120			1	1
130	8	8	9	9
140	1	1	1	1
160	1	1		
180	3	3	4	4
190	1	1		

Response is defined as ≥50% reduction of HAM-D score after DBS optimization (T2) compared to baseline.
Abbreviations: L=Left; R=Right; SD=Standard deviation.

Table S2 Overview of medication use among patients

		Non-responder T2			Responder T2		
		Baseline	*T2*	*T5*	*Baseline*	*T2*	*T5*
**Antidepressant**	*Combination*	2	5	6	0	0	0
	Single	9	7	6	4	2	2
	None	4	3	3	6	8	8
**Antipsychotic**	*Combination*	2	0	0	0	0	0
	Single	10	10	10	5	3	2
	None	3	5	5	5	7	8
**Benzodiazepine**	*Combination*	5	3	4	1	0	0
	Single	4	9	7	4	2	1
	None	6	3	4	5	8	9
**Lithium**	*Single*	2	1	1	0	0	0
	None	13	14	14	10	10	10
**Anxiolytic**	*Single*	1	1	1	0	0	0
	None	14	14	14	10	10	10
**Anti-epileptic**	*Single*	2	1	1	0	0	0
	None	13	14	14	10	10	10
**Antihistaminic**	*Single*	2	2	2	0	0	0
	None	13	13	13	10	10	10
**Opioid**	*Single*	1	2	2	0	0	0
	None	14	13	13	10	10	10
**Sympathicomimetic**	*Single*	0	0	1	1	1	1
	None	15	15	14	9	9	9

Baseline: before DBS surgery, T2: after optimization phase; T5: after second year of follow-up
